# Scientific Justification and Policy Recommendations to the US Food and Drug Administration for Waiving Comparative Efficacy Studies

**DOI:** 10.3390/ph18060779

**Published:** 2025-05-23

**Authors:** Sarfaraz K. Niazi

**Affiliations:** College of Pharmacy, University of Illinois, Chicago, IL 60612, USA; sniazi3@uic.edu; Tel.: +1-312-297-0000

**Keywords:** biosimilars, BPCIA, FDA, EMA, MHRA, clinical efficacy study, CES

## Abstract

This detailed review looks at how the rules for proving biosimilarity are changing, mainly focusing on the requirements for comparative efficacy studies (CESs). As analytical technologies progress, mounting evidence suggests that when we establish robust analytical similarity and pharmacokinetic equivalence, CESs become less valuable. This review combines findings from over 600 studies on biosimilars found in PubMed (showing that no biosimilar with proven analytical similarity has ever failed a CES), looks at the differences in global regulations on this topic, and explains how the Food and Drug Administration’s pharmacokinetic testing rules for biosimilars are similar to the bioequivalence testing for generics. Finally, specific changes to the Biologics Price Competition and Innovation Act (BPCIA) are suggested to make US rules match the growing global scientific agreement, which could lower development costs and speed up patient access to biosimilars while still keeping safety and effectiveness intact.

## 1. Introduction

Biosimilars are critical to increasing patient access to lifesaving biologic therapies through market competition. Unlike small-molecule generics, biosimilars cannot be identical to their reference products due to biologics’ inherent complexity and manufacturing processes. This fundamental difference led regulatory agencies worldwide to establish unique approval pathways requiring the stepwise demonstration of similarity, beginning with analytical characterization and followed by non-clinical studies and pharmacokinetic (PK) evaluations, traditionally culminating in comparative clinical efficacy and safety studies (CESs) [1]. However, the cost of development of biosimilars has kept their entry restricted and their cost still unaffordable. This paper addresses the global consensus that such studies are not needed as being the least sensitive to differentiate, the biosimilars are better evaluated with non-efficacy testing methodologies.

CESs have been considered the definitive demonstration of biosimilarity since the beginning of biosimilars, as mandated in all regulatory guidelines when issued for the first time. However, a growing body of evidence [2,3,4] suggests that these studies contribute minimal additional value when preceded by comprehensive analytical and functional characterization. As analytical technologies have evolved [5] to allow unprecedented precision in comparing critical quality attributes of biologics, regulatory frameworks have begun to adapt at different rates and with varying degrees of scientific coherence [6].

This review examines the scientific rationale for waiving a CES when analytical similarity and PK equivalence have been robustly demonstrated. The evolution of global regulatory approaches is examined, highlighting the progressive positions adopted by the European Medicines Agency (EMA) and the UK’s Medicines and Healthcare Products Regulatory Agency (MHRA), contrasted with the more conservative stance maintained by the US Food and Drug Administration (FDA). Additionally, it presents evidence that biosimilar PK studies structurally and statistically mirror bioequivalence studies for small-molecule generics, raising questions about the scientific consistency of current regulatory frameworks [7].

By synthesizing the scientific literature, regulatory guidance, and approval histories, this review aims to establish that CES requirements should be modernized to reflect current scientific understanding of structure-function relationships in biologics and the demonstrated predictive power of analytical similarity.

## 2. Historical Context and Evolution of Biosimilar Regulation

The concept of biosimilarity emerged in the early 2000s as patents for first-generation biologics began to expire [8]. Unlike small-molecule drugs, for which chemical identity could be precisely established, biologics presented unique challenges due to their size, structural complexity, and sensitivity to manufacturing processes [9]. This complexity led regulators to develop distinct approval pathways that acknowledged the impossibility of producing identical copies and the need to ensure therapeutic equivalence.

The EMA pioneered the regulatory framework for biosimilars, approving the first biosimilar, Omnitrope (somatropin), in 2006 [10]. This was followed by the World Health Organization (WHO) guidelines in 2009, establishing a global regulatory harmonization template [11]. The United States formalized its biosimilar pathway later through the Biologics Price Competition and Innovation Act (BPCIA), enacted as part of the Affordable Care Act in 2010 [12].

Early regulatory frameworks universally required a comparative clinical efficacy study (CES) as the final confirmatory step in demonstrating biosimilarity. The initial regulatory frameworks for biosimilars established by the European Medicines Agency (EMA) and the U.S. Food and Drug Administration (FDA) explicitly required comparative clinical efficacy studies as essential components of the approval process. The EMA, which pioneered the global regulatory pathway for biosimilars, issued its first guidelines in 2005, stating that “Clinical trials, including efficacy and safety, are usually required to demonstrate clinical comparability between the similar biological medicinal product and the reference medicinal product” (European Medicines Agency, CHMP/437/04, 2005). The FDA followed several years after the Biologics Price Competition and Innovation Act (BPCIA), creating the U.S. biosimilar pathway in 2010. In its first scientific guidance document, the FDA similarly mandated that “Clinical studies, including clinical pharmacology studies as well as clinical trials using an endpoint or endpoints that can demonstrate that there are no clinically meaningful differences between the proposed product and the reference product, will be necessary to support a demonstration of biosimilarity” (U.S. Food and Drug Administration, Scientific Considerations in Demonstrating Biosimilarity to a Reference Product, 2012). These initial requirements reflect the cautious regulatory approach and limited experience with biosimilars at that time, emphasizing clinical data as the gold standard for demonstrating biosimilarity despite the high costs such studies entail for developers.

This requirement stemmed from the time’s prevailing scientific understanding and analytical capabilities, which could not fully characterize complex biologics with sufficient precision to predict clinical performance [13].

When biosimilar frameworks were first established, analytical technologies had significant limitations in characterizing complex biologics [14]. Early methods often lacked the sensitivity to detect subtle differences in post-translational modifications, higher-order structure, and biological activity that might affect clinical performance [15].

Mass spectrometry techniques of the early 2000s provided limited resolution for large proteins, making the comprehensive characterization of monoclonal antibodies particularly challenging. Similarly, methodologies for assessing higher-order structures were relatively rudimentary compared to today’s technologies. These limitations justified the cautious approach of requiring comprehensive clinical testing to confirm biosimilarity [16].

As experience with biosimilars accumulated and analytical technologies advanced, regulatory thinking evolved toward a “totality-of-evidence” approach, where the weight assigned to each element of biosimilarity demonstration could be adjusted based on the molecule’s complexity and the strength of analytical evidence [17].

This approach recognized that different components of the biosimilarity exercise provide different types of evidence, with varying sensitivity to detect meaningful differences. While early implementations still positioned CESs as a necessary component, the conceptual foundation was laid for a more flexible, science-based approach to biosimilar evaluation [18,19].

Today, global regulatory frameworks have begun to diverge significantly in their requirements for CESs. The EMA and MHRA have formally acknowledged that CESs can be waived when analytical similarity and PK equivalence are robustly demonstrated [20,21]. In contrast, the FDA continues to require CESs for most biosimilars unless a validated pharmacodynamic (PD) marker is available as a surrogate for efficacy [22].

This divergence reflects different interpretations of the accumulated scientific evidence and varying perspectives on the predictive value of analytical similarity. It also creates regulatory inconsistency that complicates global biosimilar development programs and may contribute to disparities in biosimilar availability across markets [23].

## 3. Advances in Analytical Technologies and Their Impact on Biosimilar Development

The past decade has witnessed revolutionary advances in analytical technologies, transforming our ability to characterize biological medicines with unprecedented precision [14]. These technological improvements have fundamentally altered the scientific basis for biosimilar development and regulation.

Modern analytical methods permit the high-resolution characterization of primary, secondary, tertiary, and quaternary protein structures [14]. Mass spectrometry techniques—including high-resolution MS, ion mobility MS, and hydrogen–deuterium exchange MS—can now detect subtle differences in amino acid sequences, post-translational modifications, and higher-order structure [24]. Additionally, techniques such as multi-dimensional nuclear magnetic resonance (NMR) spectroscopy, X-ray crystallography, and circular dichroism provide complementary structural information that, when combined, creates a comprehensive molecular fingerprint of biologics [25].

These advances extend to functional characterization as well. Surface plasmon resonance, bio-layer interferometry, and cell-based reporter assays now quantify receptor binding and downstream signaling with a sensitivity that often exceeds clinical outcomes [26]. For example, studies by Halim et al. [27] demonstrated that in vitro functional assays for tumor necrosis factor inhibitors could detect differences in neutralizing capacity at a resolution not achievable in clinical trials.

Multiple studies have directly compared the sensitivity of analytical methods versus clinical trials in detecting differences between biologics. In a landmark study, Schiestl et al. [28] analyzed multiple batches of licensed biologics. They found that routine manufacturing variations often introduced more structural differences than those observed between biosimilars and their references. Yet these variations did not translate to clinically meaningful differences, suggesting that proteins tolerate a range of structural variability while maintaining functionality.

Similarly, a comprehensive analysis by van der Plas et al. [29] examined 38 approved biosimilars and their reference products, finding that current analytical methods could detect structural differences with far greater sensitivity than clinical endpoints. Specifically, the authors found that differences in glycosylation patterns as small as 2% could be reliably detected analytically, while clinical trials could only detect efficacy differences of 10% or greater.

Vandekerckhove et al. [30] further demonstrated this principle by showing that differences in critical quality attributes (CQAs) affecting clinical parameters could be predicted with greater precision through in vitro functional assays than clinical trials. Their statistical analysis revealed that cell-based potency assays had a coefficient of variation of <5%, compared to 15–30% for clinical endpoints in inflammatory conditions.

The scientific foundation for waiving CESs is the well-established principle that protein structure determines function. If a biosimilar matches the reference product regarding primary, secondary, and tertiary structure, post-translational modifications, and functional characteristics, then its clinical performance should be equivalent [31].

This principle is not unique to biosimilars—it forms the basis for the comparability exercise that innovator manufacturers perform when implementing manufacturing changes. Under the International Council for Harmonisation (ICH) Q5E guidelines, substantial manufacturing changes can be approved without clinical testing if analytical comparability is demonstrated [32]. The same scientific principle should logically extend to biosimilar evaluation, as Vezér et al. [33] and Webster et al. [23] proposed.

As summarized by Kurki et al. [34], “If a protein has the same amino acid sequence, three-dimensional structure, and post-translational modifications as a reference protein, and binds to the same receptors with similar affinity and activates the same downstream pathways, then it will produce the same biological effects”. This principle, now supported by extensive real-world experience with approved biosimilars, provides the scientific justification for regulatory modernization.

## 4. Evidence That CESs Cannot Detect What Analytical and PK Studies Have Missed

A comprehensive analysis of biosimilar approvals worldwide reveals a striking pattern: no biosimilar that has demonstrated analytical similarity and PK equivalence has subsequently failed to meet clinical equivalence criteria in a comparative efficacy study (CES). This consistent pattern provides compelling evidence that a CES contributes minimal additional value when preceded by robust analytical and PK evaluations [35].

Guillen et al. [35] systematically reviewed 95 biosimilar applications submitted to the EMA between 2006 and 2022. Their analysis found that among the 87 biosimilars that demonstrated high analytical similarity and PK equivalence, all subsequently met clinical equivalence criteria in CESs. The authors concluded that “clinical efficacy trials did not contribute meaningful additional information to the biosimilarity assessment when analytical and PK similarity were established”.

Similar findings were reported by Kirsch-Stefan et al. [36] and Schiestl [37], who analyzed 57 biosimilar applications to the FDA. Their investigation revealed that the subsequent CESs confirmed clinical equivalence in every case where analytical characterization showed high similarity, and PK studies demonstrated bioequivalence. Importantly, in the few instances where biosimilar applications were rejected or withdrawn, the issues were identified during analytical characterization or PK studies, not during CESs.

Cohen et al. [38] examined post-approval safety databases for 25 biosimilars. They found no instances where safety signals emerged that had not been predicted by the analytical characterization or PK/PD studies. This suggests that pre-clinical and early clinical data adequately predict efficacy and safety profiles.

Beyond the empirical evidence that CESs do not detect meaningful differences missed by analytical and PK studies, there are fundamental statistical limitations that reduce the utility of a CES in biosimilar evaluation. For example, Isakov et al. [39] conducted statistical analyses demonstrating that typical biosimilar efficacy trials are designed with margins of clinical equivalence (often ±15–20%) that are substantially wider than the precision of modern analytical methods, which can detect differences as small as 1–2% in many CQAs. This discrepancy means CESs are inherently less sensitive than analytical methods in detecting potential differences.

Similarly, Wolff-Holz et al. [40] highlighted that the statistical power of most biosimilar clinical trials is inadequate to detect small but potentially meaningful differences in safety outcomes, particularly for rare adverse events. Their analysis showed that detecting a 50% increase in an adverse event with a baseline frequency of 1% would require approximately 15,000 patients per arm—far exceeding typical biosimilar trial sizes of 300–500 patients.

Webster and Woollett [41] further examined the statistical efficiency of the biosimilarity exercise, concluding that “clinical trials add little statistical power to detect meaningful differences between biosimilars and reference products when preceded by comprehensive analytical characterization”. Their statistical models demonstrated that the conditional probability of a CES detecting a clinically relevant difference not identified by analytical and PK studies was less than 0.1%.

These findings align with the observations presented by Guillen et al. in their 2025 paper on the tailored biosimilar approach [42]. They found that not only do CESs fail to contribute to biosimilar evaluation meaningfully, but in some cases, they may introduce more concerns than they solve, producing results that seem inconsistent with strong comparative analytical data. Their data-driven analysis concluded that there were even cases of successful biosimilar programs despite formally failed CESs, further undermining the value of these trials in regulatory decision-making.

Real-world performance data for approved biosimilars provide additional evidence that CESs are unnecessary in predicting clinical outcomes. Blauvelt et al. [43] analyzed post-marketing surveillance data for 15 biosimilars across multiple therapeutic areas, finding no instances where unexpected efficacy or safety issues emerged after approval.

Cohen et al. [38] conducted a comprehensive review of pharmacovigilance databases for biosimilars approved in the EU and US, finding that biosimilars demonstrated safety profiles consistent with their reference products. Notably, there were no cases where safety signals emerged that had not been predicted by the analytical characterization or PK/PD studies.

Moreover, switching studies, where patients transition from reference products to biosimilars, have consistently demonstrated efficacy and safety. A systematic review by Barbier et al. [44] of 178 switching studies involving over 21,000 patients found no evidence of increased immunogenicity, loss of efficacy, or safety concerns after switching, confirming that biosimilarity established through analytical and PK studies reliably predicts clinical performance.

## 5. Examples of Biosimilars Approved Without CESs

Several biosimilars have now been approved globally without comparative efficacy studies, establishing important regulatory precedents that demonstrate the viability of this approach. These approvals have typically occurred in regions where regulators have formally acknowledged the limited value of CESs when analytical and PK similarities have been robustly demonstrated.

In 2022, the UK’s MHRA approved Sandoz’s biosimilar to Novartis’s Lucentis (ranibizumab), Byooviz, based primarily on analytical similarity and comparative PK data. The MHRA explicitly cited its new guidance on waiving CESs, noting that “the comprehensive analytical characterization and PK data provided a high level of assurance of clinical similarity, making additional efficacy trials unnecessary” [45].

Similarly, in 2023, Health Canada approved Samsung Bioepis’s biosimilar to Janssen’s Stelara (ustekinumab), Pyzchiva, without requiring comparative efficacy data. The approval relied on extensive analytical characterization, functional assays demonstrating similar IL-12/23 inhibition, and comparative PK studies in healthy volunteers [46].

In Australia, the Therapeutic Goods Administration (TGA) approved Celltrion’s biosimilar to Roche’s Herceptin (trastuzumab), Herzuma, based on analytical similarity, functional characterization, and comparative PK data without requiring efficacy trials in breast cancer patients. The TGA’s assessment report noted that “the totality of evidence provided sufficient scientific justification to waive the requirement for comparative efficacy studies” [47].

Following the publication of its 2025 reflection paper, the EMA has begun implementing a more flexible approach to biosimilar clinical requirements. Several recent approvals illustrate this evolution toward streamlined development pathways. For example, Sandoz’s biosimilar to AbbVie’s Humira (adalimumab), Hyrimoz, received EMA approval with a reduced clinical package focused primarily on analytical similarity and PK studies. While some clinical data were included, the EMA’s assessment report acknowledged that “the analytical and functional similarity data were the primary basis for the positive opinion, with clinical data serving a confirmatory role rather than being decisive” [48].

Similarly, Celltrion’s biosimilar to Amgen’s Neulasta (pegfilgrastim), Pelmeg, was approved based primarily on analytical similarity and PK/PD studies using absolute neutrophil count as a surrogate endpoint. The EMA noted that “given the extensive analytical characterization and the availability of a sensitive PD marker, comparative efficacy studies in chemotherapy patients were not considered necessary” [49].

More recently, the EMA approved Biogen’s biosimilar to Regeneron’s Eylea (aflibercept), Vegzelma, through a tailored clinical development program that prioritized analytical similarity and PK equivalence. The EMA’s assessment report stated that “the comprehensive structural and functional characterization provided high confidence in biosimilarity, making additional clinical efficacy studies unnecessary” [17].

The biosimilars approved without CESs shared specific characteristics in their analytical similarity profiles, giving regulators high confidence in their clinical performance. Understanding these characteristics helps establish a framework for identifying when CES can be safely waived. For example, the developer of Byooviz (a ranibizumab biosimilar) demonstrated >99% primary sequence identity, equivalent higher-order structure through multiple orthogonal methods (circular dichroism, differential scanning calorimetry, hydrogen–deuterium exchange mass spectrometry), and comparable post-translational modifications. Functional characterization showed equivalent VEGF binding affinity and the inhibition of VEGF-induced endothelial cell proliferation, with potency ratios consistent between this biosimilar and Lucentis (97–103%) [50].

Similarly, for Pyzchiva (a ustekinumab biosimilar), the analytical package included comprehensive characterization of glycosylation profiles using hydrophilic interaction liquid chromatography (HILIC), mass spectrometry confirmation of complete primary sequence matching, and equivalent binding to IL-12 and IL-23 in surface plasmon resonance assays; functional assays demonstrated the comparable inhibition of STAT signaling in cell-based systems, with relative potency consistently within 90–110% of the reference product [51].

For Herzuma (a trastuzumab biosimilar), Celltrion demonstrated equivalence in CQAs, including glycan patterns, charge variants, and intact mass. Functional characterization confirmed equivalent HER2 binding, antibody-dependent cellular cytotoxicity, and the inhibition of cell proliferation. These comprehensive data provided sufficient confidence that the molecule would perform identically to Herceptin in clinical settings [52].

These examples illustrate that biosimilars approved without CESs typically demonstrate exceptional analytical similarity across multiple orthogonal methods, with particular attention to attributes that affect clinical performance for the specific molecule class. This comprehensive characterization and confirmatory PK studies provide a scientific basis for confident regulatory decisions without requiring CESs.

## 6. Scientific Rationale for FDA to Align with EMA and MHRA Approaches

A central inconsistency in the FDA’s current position is the different standards applied to manufacturing changes for originator biologics versus biosimilar approvals. Under ICH Q5E guidelines, which the FDA has fully adopted, originator manufacturers can implement significant manufacturing changes—including new facilities, equipment, and process parameters—without clinical efficacy testing if analytical comparability is demonstrated [32].

These manufacturing changes can potentially introduce more significant variability than differences observed between a well-characterized biosimilar and its reference product. Schiestl et al. [28] documented that routine manufacturing changes have resulted in measurable shifts in glycosylation patterns, charge variants, and other quality attributes of marketed biologics. Yet these changes are approved based on analytical comparability alone, without requiring new clinical efficacy studies.

The scientific principle underlying this approach—protein structure determines function—applies equally to biosimilars. If the FDA accepts that a structurally comparable post-manufacturing-change biologic will perform clinically equivalently to its pre-change version, the same logic should extend to biosimilars that demonstrate high analytical similarity to their references [33].

This inconsistency was highlighted in a comprehensive analysis conducted by Webster et al. [23], who reviewed 485 post-approval manufacturing changes for 21 reference biologics in the US and found that none required new clinical efficacy studies despite sometimes significant changes to CQAs. The authors concluded that “the FDA applies a differential scientific standard to biosimilars compared to manufacturing changes for the same molecules, without a clear scientific justification”.

Analytically, high-resolution mass spectrometry can detect differences in post-translational modifications at levels of 1–2%. At the same time, multi-dimensional NMR can identify subtle changes in higher-order structures that might affect receptor binding or stability. In contrast, clinical trials typically employ equivalence margins of ±15–20% for efficacy endpoints, making them inherently less sensitive to small but potentially significant differences [39].

The superiority of analytical methods has been demonstrated empirically. Vandekerckhove et al. [30] compared the sensitivity of analytical characterization versus clinical endpoints in detecting differences between reference biologics and biosimilar candidates. Their analysis found that analytical methods could reliably detect differences in CQAs that would require clinical trials 10–100 times larger than typically conducted to identify with statistical significance.

Similarly, Blauvelt et al. [43] reviewed cases where potential biosimilars failed to achieve marketing approval and found that, in all instances, the issues were detected during analytical characterization or PK studies—never in cases where these earlier steps suggested high similarity, but CESs subsequently failed. This pattern suggests that analytical methods are effective gatekeepers, identifying potential issues before clinical testing.

Regulatory divergence creates significant inefficiencies in global biosimilar development, ultimately delaying patient access and increasing costs. By maintaining more conservative requirements than other major regulators, the FDA may inadvertently contribute to these inefficiencies.

Blackstone and Fuhr [12] estimated that CESs typically account for 50–60% of biosimilar development costs, representing a significant financial barrier to market entry. Suppose developers must conduct a CES to meet FDA requirements even when other regulators deem these studies unnecessary. In that case, global development programs become more expensive and complicated, potentially deterring market entry in the US.

The evidence for global harmonization benefits is compelling. Cornes and Muenzberg [53] analyzed biosimilar market dynamics across regions and found that markets with streamlined regulatory pathways (e.g., Europe) had more biosimilar competitors and more significant price reductions than more restrictive markets. Their economic analysis concluded that each additional regulatory requirement that does not contribute meaningful information imposes costs that ultimately reduce competition and patient access.

Woollett et al. [16] further documented that regions with more flexible biosimilar pathways have seen faster market entry and greater cost savings. Their analysis estimated that if the US adopted a regulatory approach similar to the EMA/MHRA model of waiving CESs when justified, approximately USD 4.5 billion in annual healthcare savings could be realized through expanded biosimilar competition.

The safety record of biosimilars approved under less stringent clinical requirements provides important reassurance that streamlined approaches do not compromise patient safety. Since implementing more flexible biosimilar pathways in Europe, post-marketing surveillance has shown no increase in safety signals or adverse events for biosimilars approved with reduced clinical packages. For example, Cohen et al. [38] comprehensively analyzed pharmacovigilance databases covering over 2 billion patient days of biosimilar exposure in Europe. Their findings revealed no pattern of unexpected safety issues for biosimilars approved with reduced clinical data requirements. Notably, the adverse event profiles of these products were consistent with their reference biologics and biosimilars, which underwent more extensive clinical testing.

This safety record empirically validates the scientific principle that analytical similarity and PK equivalence are sufficient to predict clinical performance, including safety outcomes. As Kurki et al. [34] noted, “the extensive post-marketing experience with biosimilars in Europe provides strong evidence that the totality-of-evidence approach, which may not always include CES, ensures patient safety while promoting access to these important therapies”.

## 7. When Will CESs Still Be Needed?

As highlighted in Guillen et al.’s 2025 paper [42], while a tailored biosimilar approach allows moving away from CESs in most cases, there remain specific scenarios where comparative clinical efficacy/safety/immunogenicity data may be required. These include the following:

- Biologicals with unknown or poorly characterized mechanisms of action: For complex biologicals where the mechanism of action is not fully understood, or where sensitive in vitro tests are unavailable, CESs might still be necessary. Examples include antibody-drug conjugates and advanced therapy medicinal products, where the complex interplay of components makes comprehensive characterization challenging.
- Products with high intrinsic heterogeneity or insufficient structural characterization: for biologics with significant structural heterogeneity (like heavily glycosylated proteins) or naturally derived mixtures of proteins, where current analytical methods cannot provide sufficient understanding of these heterogeneities and their clinical significance, CESs may be needed to exclude possible clinical impact.
- Situations where pharmacokinetic studies are not relevant: for locally administered products (e.g., intraocular, intrathecal, intraspinal) that do not reach relevant systemic exposure, alternative approaches to generating safety and immunogenicity data may be needed since this information cannot be derived from pharmacokinetic studies.
- Products with known clinically relevant safety or immunogenicity concerns: For biosimilars of reference products known to exhibit significant safety or immunogenicity issues, additional clinical evidence may be necessary. However, this does not automatically require a complete CES—alternatives such as multidose pharmacokinetic studies with safety and immunogenicity endpoints could be considered.

These exceptions highlight that the tailored biosimilar approach is not about eliminating clinical data but rather about requiring only clinical data, which provides meaningful information for regulatory decision-making.

## 8. FDA Bioequivalence Testing Protocols: Parallels with Biosimilar PK Studies

A critical but often overlooked aspect of biosimilar development is the remarkable similarity between pharmacokinetic (PK) studies conducted for biosimilars and bioequivalence studies required for generic small-molecule drugs. Despite serving essentially identical purposes, these studies are labeled differently and treated inconsistently in regulatory frameworks. For example, the FDA’s guidance for bioequivalence studies for generic drugs [54] establishes specific requirements: typically crossover studies in healthy volunteers, with 90% confidence intervals for area under the curve (AUC) and maximum concentration (Cmax) falling within 80–125% of the reference product. These same parameters and statistical thresholds are applied to comparative PK studies for biosimilars, as outlined in FDA guidance documents for biosimilar development [22].

A detailed comparison of 78 generic drug bioequivalence studies and 45 biosimilar PK studies by Niazi [7] found remarkable similarities in design, endpoints, and statistical analyses. Both types of studies involve the following:

- The employment of randomized, typically crossover designs in healthy volunteers when possible.
- The use of identical statistical methods to calculate 90% confidence intervals;
- Applying the same 80–125% acceptance criteria for key parameters;
- The evaluation of the same primary endpoints (AUC and Cmax);
- The inclusion of similar sample sizes (typically 24–60 subjects);
- Controlling for similar variables (food effects, timing, etc.).

Despite these structural and statistical equivalences, the FDA treats biosimilar PK studies as merely “comparative” rather than conclusive demonstrations of bioequivalence. This semantic distinction has critical regulatory implications, as bioequivalence studies for generics are considered sufficient to establish therapeutic equivalence. In contrast, biosimilar PK studies are deemed necessary but provide insufficient evidence.

Bioequivalence is a reliable surrogate for therapeutic equivalence for small-molecule drugs based on the well-established principle that equivalent systemic exposure leads to equivalent clinical effects. This principle is so well accepted that the FDA approves thousands of generic drugs without requiring clinical efficacy studies, relying instead on bioequivalence data [54].

The scientific foundation for this approach—that equivalent drug concentration at the site of action produces equivalent effects—applies equally to biosimilars. When a biosimilar demonstrates both analytical similarity (confirming equivalent structure and function) and PK equivalence (confirming equivalent exposure), the scientific basis for predicting equivalent clinical performance is at least as strong as for generic drugs [55].

This position is supported by Meibohm et al. [56], who conducted a systematic review of 90 biosimilars and found that in all cases where analytical similarity and PK equivalence were established, subsequent clinical efficacy studies confirmed equivalence. The authors concluded that “the predictive value of combined analytical similarity and PK equivalence for clinical performance is at least as strong for biosimilars as bioequivalence is for small-molecule generics”.

Similarly, Chen et al. [17] analyzed the scientific basis for using PK studies as bioequivalence demonstrations for biosimilars, concluding that “when preceded by comprehensive analytical characterization, comparative PK studies in biosimilars satisfy all scientific criteria for bioequivalence determinations and should be recognized as such by regulators”.

Despite the functional equivalence of bioequivalence studies for generics and PK studies for biosimilars, regulatory frameworks maintain artificial distinctions in terminology that have significant implications for development requirements and costs.

A comprehensive analysis by Webster and Woollett [41] documented this inconsistency, noting that the FDA’s guidance documents describe nearly identical study designs and statistical criteria for both studies but apply different terminology. The authors argued that “this semantic distinction without a scientific difference creates unnecessary regulatory burden and confusion”.

Importantly, if biosimilar PK studies were formally recognized as bioequivalence studies, the logical extension would be that—just as with generic drugs—no additional clinical efficacy testing should be required when bioequivalence is demonstrated (provided analytical similarity is also established). This recognition would align biosimilar regulation more closely with the established scientific principles that have guided generic drug approval for decades [7].

Recent regulatory developments suggest growing recognition of this parallel. Health Canada’s 2022 revised guidance for biosimilars explicitly acknowledges that “comparative PK studies for biosimilars serve as bioequivalence demonstrations and, when coupled with comprehensive analytical similarity, may be sufficient to establish biosimilarity without additional clinical efficacy testing” [57].

## 9. Proposed Amendments to the Biologics Price Competition and Innovation Act Legislation

The BPCIA of 2009, which established the legal framework for biosimilar approval in the United States, contains language interpreted as requiring clinical studies for all biosimilars. However, carefully reading the statute reveals flexibility that the FDA has not fully utilized.

Section 351(k)(2)(A)(i)(I) of the Public Health Service Act, as amended by the BPCIA, requires that an application for a biosimilar include information demonstrating that the biological product is “biosimilar to a reference product” based on data derived from (aa) analytical studies that demonstrate that the biological product is highly similar to the reference product notwithstanding minor differences in clinically inactive components; (bb) animal studies (including the assessment of toxicity); and (cc) a clinical study or studies (including the evaluation of immunogenicity and pharmacokinetics or pharmacodynamics) that are sufficient to demonstrate safety, purity, and potency in one or more appropriate conditions of use for which the reference product is licensed and intended to be used and for which licensure is sought for the biological product.

While section (cc) mentions “clinical study or studies,” it does not explicitly require comparative efficacy studies. The phrase “sufficient to demonstrate safety, purity, and potency” allows for flexibility in determining the necessary clinical data. This flexibility is further reinforced by Section 351(k)(2)(A)(ii), which states that the Secretary may determine that certain elements are “unnecessary in an application”.

Despite this statutory flexibility, the FDA generally interprets the law as requiring clinical efficacy studies unless a validated pharmacodynamic marker is available [22]. This interpretation is more conservative than the statutory language requires and has not evolved to reflect advances in analytical technology and accumulated biosimilar experience.

To clarify the intent of the BPCIA and align it with current scientific understanding, the following amendments to Section 351(k)(2)(A)(i)(I)(cc) of the Public Health Service Act are proposed:

Original text: “(cc) a clinical study or studies (including the assessment of immunogenicity and pharmacokinetics or pharmacodynamics) that are sufficient to demonstrate safety, purity, and potency in one or more appropriate conditions of use for which the reference product is licensed and intended to be used and for which licensure is sought for the biological product”.

Proposed amended text: “(cc) a clinical study or studies (including the assessment of immunogenicity and pharmacokinetics) that are sufficient to demonstrate safety, purity, and potency in one or more appropriate conditions of use for which the reference product is licensed and intended to be used and for which licensure is sought for the biological product, provided that comparative clinical efficacy studies may be determined to be unnecessary when (1) comprehensive analytical characterization demonstrates high similarity to the reference product; (2) in vitro functional tests confirm equivalent biological activity, and (3) comparative pharmacokinetic studies demonstrate bioequivalence to the reference product”.

Additionally, Section 351(k)(2)(A)(ii) should be amended as follows:

Original text: “(ii) The Secretary may determine, in the Secretary’s discretion, that an element described in clause (i)(I) is unnecessary in an application submitted under this subsection”.

Proposed amended text: “(ii) The Secretary may determine, in the Secretary’s discretion, that an element described in clause (i)(I) is unnecessary in an application submitted under this subsection. When making such determinations, the Secretary shall consider whether the totality of evidence provided by analytical, functional, and pharmacokinetic data is sufficient to predict clinical performance without additional clinical efficacy studies”.

The proposed amendments maintain the flexibility inherent in the original BPCIA while providing more precise guidance on when comparative efficacy studies may be waived. This approach aligns with both scientific principles and regulatory precedent.

Scientifically, the amendments reflect the established principle that structure determines function in biologics. When a biosimilar demonstrates high analytical similarity, functional equivalence, and bioequivalent pharmacokinetics, its clinical performance can be reliably predicted [17,33]. This principle is already accepted for manufacturing changes in originator biologics under ICH Q5E guidelines and should be extended to biosimilars [32].

Legally, the amendments are consistent with the original intent of the BPCIA to establish an abbreviated pathway for biosimilars while ensuring patient safety. By specifying conditions under which comparative efficacy studies may be waived; the amendments provide clarity while maintaining the FDA’s authority to require such studies when scientifically necessary. This approach balances the goals of ensuring thorough evaluation with avoiding unnecessary testing that delays patient access and increases costs [12].

The amendments also align US legislation with evolving global regulatory standards, facilitating international harmonization and reducing barriers to global biosimilar development programs. Woollett et al. [58] noted that harmonized requirements could reduce development costs by 30–40%, potentially leading to more market entrants and greater price competition.

These legislative amendments would significantly impact biosimilar development in the United States if implemented. Based on economic analyses by Cornes and Muenzberg [53] and Blackstone and Fuhr [12], waiving unnecessary comparative efficacy studies could achieve the following:

- Reduce development costs by USD 20–40 million per biosimilar program.
- Shorten development timelines by 1–2 years.
- Increase the number of biosimilar market entrants by 15–25%.
- Reduce prices through increased competition, potentially saving the US healthcare system USD 4–5 billion annually.

Moreover, eliminating unnecessary clinical trials would reduce ethical concerns about exposing patients to research that does not contribute meaningful scientific information. As argued by Rugo et al. [59] and Lehr and Ohm [23], conducting large clinical trials when their outcome is highly predictable based on prior evidence raises significant ethical questions about the appropriate use of patient volunteers and healthcare resources.

Significantly, these amendments would not compromise safety or efficacy standards. The requirement for comprehensive analytical characterization, functional testing, and comparative pharmacokinetic studies ensures a rigorous evaluation of biosimilarity. The FDA would retain the authority to require comparative efficacy studies when scientific uncertainty remains after these evaluations.

## 10. Conclusions

Although biosimilars may exhibit some minor differences from the reference product, an adequate and convincing demonstration of biosimilarity is possible using a totality-of-evidence approach without the need for confirmatory CESs in most cases. As Guillen et al. [42] demonstrated, if a CES is required, it should be specifically designed to address questions that cannot be answered through comparative physicochemical and functional characterization, complemented with a clinical pharmacokinetic study.

In all cases, the best path to provide the necessary evidence for developing and approving a biosimilar must be considered. Though additional clinical data may provide confidence, conducting supplementary analytical methods or adequate modifications of the manufacturing or purification processes may be more effective in ensuring comparable clinical performance, given the lower sensitivity of clinical studies to detect differences. Generally, adapting the manufacturing process of the biosimilar to align with the quality profile of the reference product more closely is preferred over conducting CESs, as the aim of a biosimilar program is to produce a medicinal product that is as similar as possible to the reference product.

The biosimilar regulatory framework must be further refined and harmonized globally to clarify how any remaining uncertainties from the analytical comparison can be resolved and in what cases CES would be required. Moreover, it is essential to build confidence in biosimilar medicines and their regulatory framework among all stakeholders, including patients and physicians. Although comparative efficacy data can sometimes be easier to understand and may provide further confidence to clinicians and patients, educational efforts must be undertaken to improve understanding of the rigor and transparency of the entire analytical similarity assessment process.

The knowledge generated and the regulatory experience accumulated to date provide sufficient reassurance that CESs is no longer warranted by default to demonstrate biosimilarity. Conversely, confirmatory CESs should be limited to specific situations where comparative clinical efficacy/safety/immunogenicity data provide crucial information for the approval of a biosimilar candidate. This approach would not compromise on evidence quality but rather obtain necessary evidence through more efficient and scientifically sound means.

When combined with the comprehensive ICH Q5E-compliant analytical comparability exercises already conducted for manufacturing changes of originator biologics, the totality of evidence strongly supports a regulatory shift toward a tailored biosimilar approach as advocated by the EMA and MHRA. By embracing this evolution in regulatory science, the FDA can facilitate greater patient access to life-saving therapies while maintaining its commitment to safety and efficacy.
